# Defining behavioral and molecular differences between summer and migratory monarch butterflies

**DOI:** 10.1186/1741-7007-7-14

**Published:** 2009-03-31

**Authors:** Haisun Zhu, Robert J Gegear, Amy Casselman, Sriramana Kanginakudru, Steven M Reppert

**Affiliations:** 1 Department of Neurobiology, University of Massachusetts Medical School, Plantation Street, Worcester, MA 01605, USA

## Abstract

**Background:**

In the fall, Eastern North American monarch butterflies (*Danaus plexippus*) undergo a magnificent long-range migration. In contrast to spring and summer butterflies, fall migrants are juvenile hormone deficient, which leads to reproductive arrest and increased longevity. Migrants also use a time-compensated sun compass to help them navigate in the south/southwesterly direction en route for Mexico. Central issues in this area are defining the relationship between juvenile hormone status and oriented flight, critical features that differentiate summer monarchs from fall migrants, and identifying molecular correlates of behavioral state.

**Results:**

Here we show that increasing juvenile hormone activity to induce summer-like reproductive development in fall migrants does not alter directional flight behavior or its time-compensated orientation, as monitored in a flight simulator. Reproductive summer butterflies, in contrast, uniformly fail to exhibit directional, oriented flight. To define molecular correlates of behavioral state, we used microarray analysis of 9417 unique cDNA sequences. Gene expression profiles reveal a suite of 40 genes whose differential expression in brain correlates with oriented flight behavior in individual migrants, independent of juvenile hormone activity, thereby molecularly separating fall migrants from summer butterflies. Intriguing genes that are differentially regulated include the clock gene *vrille *and the locomotion-relevant *tyramine beta hydroxylase *gene. In addition, several differentially regulated genes (37.5% of total) are not annotated. We also identified 23 juvenile hormone-dependent genes in brain, which separate reproductive from non-reproductive monarchs; genes involved in longevity, fatty acid metabolism, and innate immunity are upregulated in non-reproductive (juvenile-hormone deficient) migrants.

**Conclusion:**

The results link key behavioral traits with gene expression profiles in brain that differentiate migratory from summer butterflies and thus show that seasonal changes in genomic function help define the migratory state.

## Background

Eastern North American monarch butterflies (*Danaus plexippus*) undergo a spectacular fall migration during which they travel distances up to ~4000 km to reach their overwintering grounds in central Mexico [1]. In contrast to spring and summer butterflies, fall migrants are juvenile hormone (JH) deficient, which leads to reproductive arrest (diapause), increased longevity, and increased abdominal fat stores [2,3]. Fall migrants also use a time-compensated sun compass to help them navigate in the south/southwesterly direction [4-6]. Reproductive quiescence persists at the overwintering areas in Mexico until spring, when the butterflies break diapause, become reproductively competent, mate, and fly northward to lay fertilized eggs on newly emerged milkweed plants in the southern United States [7,8].

The migrant offspring give rise to three to four successive generations of reproductively active butterflies that repopulate the northern range of their habitat. It is unclear whether the successive generations of spring and summer butterflies have oriented flight activity to the north and/or whether they are following the progressive northerly increase in milkweed abundance, while avoiding undue heat stress that would occur if they remained in the southern United States throughout the summer [7]. The late-July/early-August generations of summer butterflies, some of whose offspring become fall migrants, appear to be the best example of butterflies that do not exhibit oriented flight behavior [9,10]. However, the precise type of flight behavior that the summer monarchs actually manifest has not been rigorously examined. It is also unclear whether JH deficiency and the accompanying reproductive quiescence are required for ongoing time-compensated sun compass orientation in fall migrants.

We recently developed a brain expressed sequence tag (EST) resource for monarch butterflies that likely represents ~50% of genes in the monarch genome [11]. Using high-density microarrays of the 9417 unique cDNA sequences in the EST resource, a blueprint of gene expression patterns can be compared and contrasted between different conditions that may help define the molecular substrates that characterize the summer and migratory states.

Here we show that increasing JH activity to induce summer-like reproductive development in fall migrants did not alter directional flight behavior or its time-compensated orientation, as monitored in a flight simulator. Summer butterflies, on the other hand, uniformly failed to exhibit directional, oriented flight. Microarray analysis revealed 40 JH-independent genes whose differential expression in brain correlated with directional flight behavior in fall migrants. Moreover, we have identified 23 JH-dependent genes in brain, which separate reproductive from non-reproductive butterflies. These data provide an unprecedented foray into the genomic regulation of migratory behaviors in monarch butterflies.

## Results and discussion

### Increased juvenile hormone activity in migrants does not disrupt directed flight or time-compensated orientation

Because several aspects of migratory behavior are a consequence of continued JH deficiency, for example, reproductive quiescence and increased longevity [2,3], we examined whether the oriented flight behavior characteristic of fall migrants also depends on persistent JH insufficiency. This was evaluated by increasing JH activity with the potent JH analog methoprene [12] and then monitoring the effect on reproductive state and time-compensated flight orientation. Preliminary studies showed that the topical treatment of migrants with 200 μg of methoprene on day 1 and day 3 consistently led to summer-like reproductive development in both sexes by day 14, while vehicle (control) applications of acetone did not (data not shown; see below).

Both methoprene- and vehicle-treatment groups were housed indoors in either a 12 hr light-12 hr dark (LD) cycle that was timed to coincide with the prevailing lighting conditions or a 6 hr-delayed LD cycle. These two lighting cycles, which differed in their timing relative to each other, were used to test whether flight orientation was time compensated, because altering the timing of the daily light-dark cycle should cause predictable changes in the direction the butterflies fly, if time compensation is operable. For example, the 6-hr delay in LD should cause a clockwise shift in orientation of 72° to 120°, relative to the non-shifted LD group, if flight direction is fully time compensated; the degree of the shift expected depends on how rapidly the sun's azimuth varies during the time of day the studies were performed, which was 12° to 20° per hour for the current studies.

**Figure 1:**
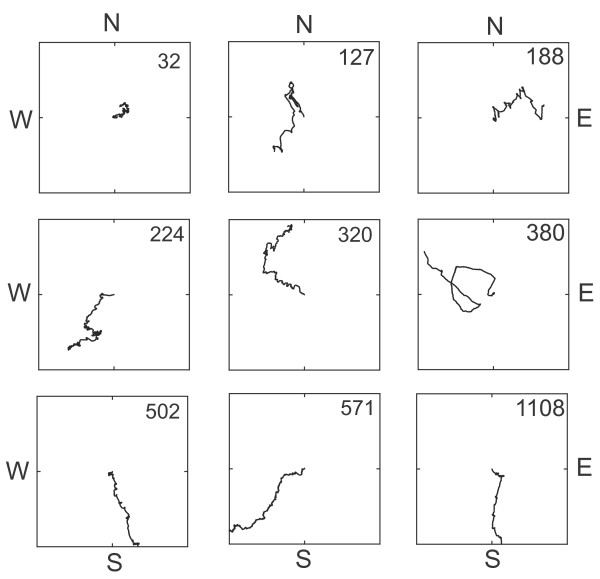
**Relationship between virtual flight path and *Z*-score value**. To obtain *Z*-scores (shown upper right of each graph), flight data for each butterfly tested in the flight simulator were analyzed using a Rayleigh test. *Z*, which is the critical value for the Rayleigh test, is calculated from the following formula: *Z *= *nr*^2^, where *n *is the number of observations and *r *is the magnitude of the mean vector. Only butterflies with a *Z*-score 500 or above have a flight path that shows clear directionality. Virtual flight paths were constructed by starting in the center of the square and plotting each direction interval consecutively as one unit length [5].

**Figure 2:**
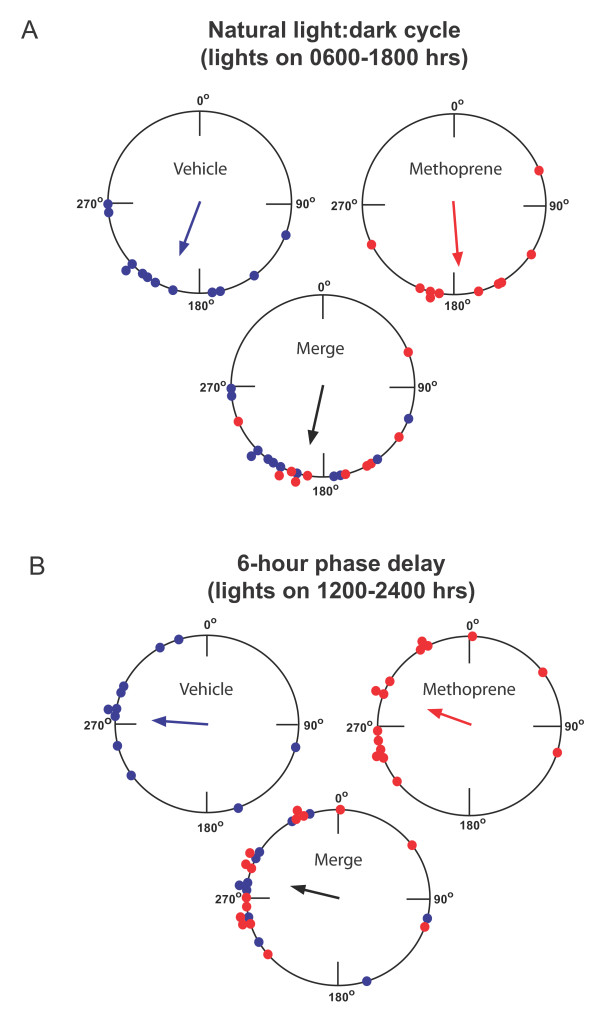
**Reproductive migrants show time-compensated sun compass orientation**. (A) Vehicle- and methoprene-treated migrants housed under normal fall conditions show a flight orientation in the south/southwesterly direction. Migrants were housed in a light-dark cycle with lights on from 0600 to 1800 hours EST with a temperature cycle of 23°C during light-12°C during dark before being tested outdoors in a flight simulator. The migrants were flown between 1230 and 1530 hours from 19 September to 15 October 2007. The large circles represent the 360° of possible direction (0° = north). The small solid circles on the perimeter represent the mean orientation of individual butterflies. Blue, vehicle-treated migrants; red, methoprene-treated migrants; merge, combined data. The arrow indicates the mean vector, and the length of the arrow represents the strength (*r *value). (B) Vehicle- and methoprene-treated migrants housed under a 6-hr phase delayed lighting cycle show a flight orientation in the west/northwesterly direction. Migrants were housed in light-dark cycle with lights on from 1200 to 2400 hours EST with a temperature cycle of 23°C during light-12°C during dark before being tested outdoors in a flight simulator. The migrants were flown between 1230 and 1530 hours from 19 September to 15 October 2007. The large circles represent the 360 of possible direction (0° = north). The small solid circles on the perimeter represent the mean orientation of individual butterflies. Blue, vehicle-treated migrants; red, methoprene-treated migrants; merge, combined data. The arrow indicates the mean vector, and the length of the arrow represents the strength (*r *value).

Fourteen days after the first methoprene treatment, butterflies housed in either LD or the 6-hr delayed LD cycle were tethered, and over the next 5 days individual flight direction and group orientation were examined in butterflies flown outdoors in a flight simulator. Of 62 migrants that flew continuously for 5 to 10 min in the simulator, 48 individuals (77% of total) flew directionally, which was defined as flying with a *Z*-score ≥ 500 (Figure 1); these directional migrants comprised the four groups that were evaluated for the time-compensated orientation analysis (Figure 2).

Regardless of treatment (methoprene or vehicle), group analyses showed that the directional fall migrants manifested time-compensated flight orientation (Figure 2). Both treatment groups housed under prevailing LD conditions were oriented significantly in the south/southwesterly direction (Figure 2A); vehicle-treated migrants had an orientation vector (α) of 202.6° (*n *= 12, *r *= 0.714, *p *= 0.001) (Figure 2A, upper left, small blue circles), similar to what we have reported before for untreated migrants [4], and methoprene-treated migrants had an α of 173° (*n *= 10, *r *= 0.713, *p *= 0.004) (Figure 2A, upper right, small red circles). The mean flight orientation did not differ between vehicle- and methoprene-treated migrants (*p *= 0.18; Watson-Williams F-test) and the combined α was 189.2° (*n *= 22, *r *= 0.69, *p *< 0.00001) (Figure 2A, lower, merge).

Both treatment groups housed under the 6 hr-delayed LD cycle were oriented significantly in the west/northwesterly direction (Figure 2B); vehicle-treated migrants had an α of 276.9° (*n *= 11, *r *= 0.58, *p *= 0.021) (Figure 2B, small blue circles), and methoprene-treated migrants had an α of 291.6° (*n *= 15, *r *= 0.566, *p *= 0.006) (Figure 2B, small red circles). Again, the mean flight orientation did not differ between vehicle- and methoprene-treated migrants (*p *= 0.573; Watson-Williams F-test) and the combined α was 285.3° (*n *= 26, *r *= 0.567, *p *= 0.0001) (Figure 2B, merge).

**Figure 3:**
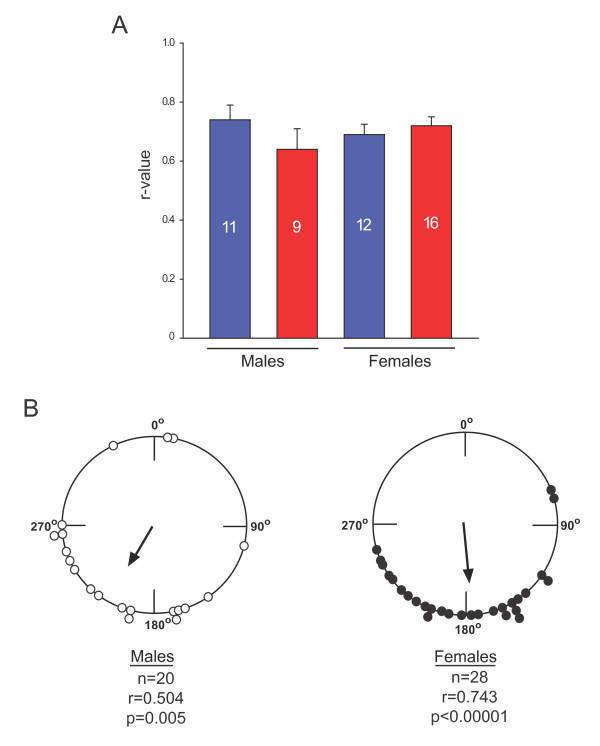
**Flight direction and orientation do not differ between male and female migrants**. A. *R*-values of flight direction were similar between treatment groups (vehicle = blue; methoprene-treated = red) and between sexes. B. Mean orientation of all directional butterflies did not differ between males and females (*p *> 0.05). The data were standardized to orientation relative to LD for both the LD and the 6-hr phase delayed LD groups to directly compare orientation of all the animals of each sex used for the studies in Figure 2.

The direction and magnitude of the group orientation difference between the merged LD and 6 hr-shifted LD groups (a clockwise shift of 96.1°; *p *< 0.00001, Watson-Williams F-test) are those expected of a time-compensated sun compass that has been delayed by 6 hrs (compare merged data in lower rows of Figure 2A and 2B). There were no significant sex differences in either the degree of individual directionality or group orientation of the migrants tested (Figure 3).

**Figure 4:**
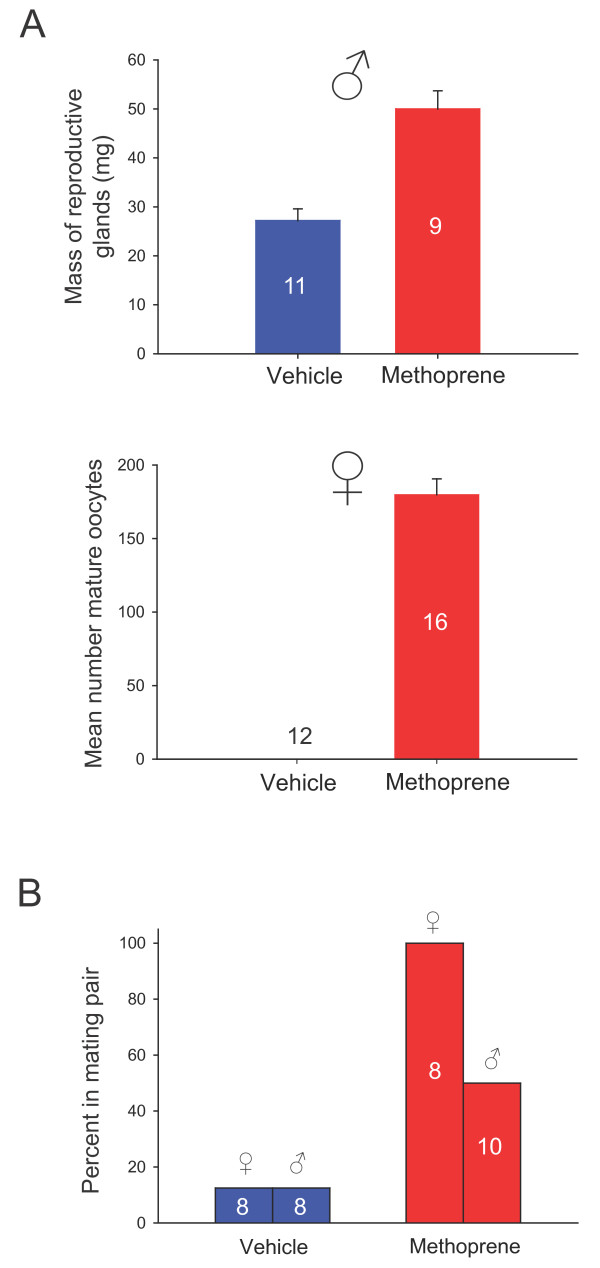
**Methoprene increases reproductive organ development and mating behavior in fall migrants**. (A) Reproductive development is increased in fall migrants following methoprene treatment. Numbers represent the animals examined. Postmortem reproductive development was assessed for all migrants depicted in Figure 2; vehicle-treated migrants (blue bars) and methoprene-treated migrants (red bars). Reproductive development of male migrants (upper panel) was quantified by extracting and weighing reproductive glands (tubular gland and ejaculatory duct), while reproductive development of female migrants (lower panel) was quantified by counting all mature oocytes (that is, those with well-defined ridged chorion). (B) Mating behavior was increased in vehicle-treated migrants (blue bars) compared to methoprene-treated migrants (red bars). Numbers represent the animals tested. To assess mating behavior, male and female butterflies from each group were placed together in flight cages under high intensity light of 2.6 × 10^5 ^photons/cm^2^/s during the light period of LD at 25°C, and the number of animals in a mating pair was recorded over a 2-day period.

Postmortem analysis of the oriented butterflies revealed that the methoprene-treated male and female migrants all had activated reproductive systems; male reproductive organ weights were almost doubled compared with the vehicle group (*p *< 0.0001), and >100 mature oocytes were found in the methoprene-treated females (Figure 4A). Furthermore, all methoprene-treated females and 50% of the methoprene-treated males examined exhibited reproductive behavior by forming mating pairs when exposed to high-intensity light and increased temperature (25°C) from day 14 to 16 after the start of methoprene treatments; this behavior was rarely observed in the vehicle-treated animals exposed to the same conditions (Figure 4B).

These data show that individual fall migratory monarchs uniformly manifest directed flight and as a group show robust time-compensated sun compass orientation even when their reproductive systems are activated (at the morphological and behavioral levels) by JH analog treatment. Although JH deficiency may be involved in the induction of directional flight and proper sun compass orientation, it is not required for their maintenance.

### Summer butterflies uniformly fail to show directed, oriented flight behavior

Although it has been reported that 'summer' monarchs do not exhibit oriented flight [9,10], until now this has not been evaluated in a flight simulator in which both individual directionality and group orientation can be assessed (see below). We tested these parameters in wild-caught summer butterflies captured in western Massachusetts (latitude 42°59'N) between 20 July and 10 August 2008 and housed indoors in a light-dark cycle that was timed to coincide with the prevailing lighting conditions. These butterflies were reproductive, as most were found in mating pairs while held in screened cages outdoors prior to being flown in a flight simulator. Moreover, fall migrants typically are not found at this locale until after 1 September.

**Figure 5:**
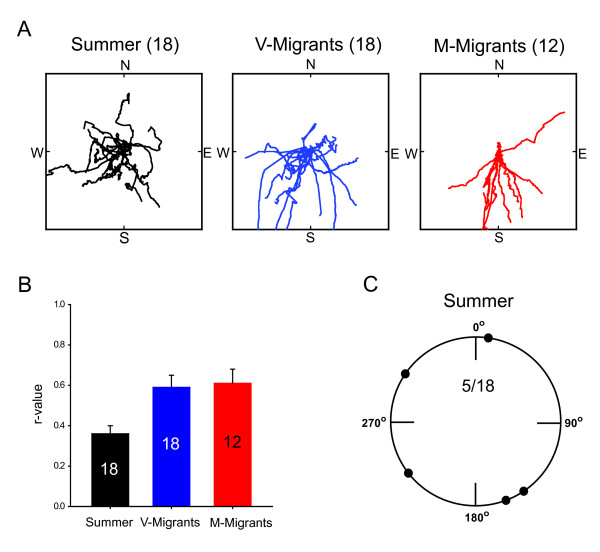
**Reproductive summer butterflies fail to consistently show directional flight**. (A) Virtual flight paths of the individual summer butterflies tested (*n *= 18, black lines), vehicle-treated migrants (V-Migrants; *n *= 18, blue lines; 12 directional [from Figure 2A]/6 non-directional) and methoprene-treated migrants (M-Migrants; *n *= 12, red lines; 10 directional [from Figure 2A]/2 non-directional) flown in flight simulator. The summer butterflies were housed under simulated summer conditions in a light-dark cycle with lights on from 0430 to 1900 hours EST at 20°C for at least three days before being tested outdoors in a flight simulator (flown between 1230 and 1530 hours). Virtual flight paths were constructed by starting in the center of the square and plotting each direction interval consecutively as one unit length [5]. (B) *R*-values for individual virtual flight paths shown in (A). *R*-values differed among summer, vehicle-treated migrants and methoprene-treated migrants (*p *= 0.005). Tukey's pairwise comparisons revealed that summer butterflies had lower *r*-values than vehicle- and methoprene-treated migrants. (C) Flight orientation of summer butterflies. Of the 18 summer butterflies flown in the flight simulator, only five showed significant directional flight. The large circles represent the 360° of possible direction (0° is north), with the small solid circles on the perimeter representing mean orientation for an individual flight.

In marked contrast to fall migrants, only 5 of 18 summer butterflies (27.8%) that flew outdoors continuously for 5 to 10 min in the flight simulator flew directionally (with *Z*-scores ≥ 500). This lack of directional flight among individuals was apparent on inspection of the constructed virtual flight paths of the summer butterflies compared with the individual flight paths of both the methoprene-treated and vehicle-treated migrants housed in LD (Figure 5A). We also compared the *r*-values for individual virtual flight paths among the three groups (Figure 5B), because *r*-values are a measure of angular dispersion and range from 0 to 1, in which 0 represents complete dispersion of the data and 1 represents all of the data concentrated in the same direction. The *r*-values differed significantly among the three groups (*p *= 0.005), and the summer butterflies had significantly lower *r*-values than those in the two migrant groups. The five summer animals with directional flight were not oriented significantly in any one direction as a group (*p *> 0.05) (Figure 5C). There were no significant sex differences in either the directionality of flight among individuals or group orientation in the summer butterflies tested (sex ratio was 1:1 within the group; six males and seven females were non-directional; three males and two females were directional).

These data show that the majority of individual mid- to late-summer butterflies exhibit non-directional flight behavior. Although the numbers were small, the data also suggest that as a group those butterflies that were directional were not significantly oriented, as previously suggested [9,10].

### Gene expression profiles in brain correlate with oriented flight behavior in fall migrants

Our behavioral data in migrants suggest that the regulation of directed flight behavior in individuals and group orientation are separable from reproductive state (Figures 2 and 4). It consequently seemed possible that there might be a set of genes that regulates oriented flight behavior in migrants that is independent of the JH pathway. We therefore performed microarray analysis to determine whether there are differentially expressed genes between summer butterflies and fall migrants, irrespective of their reproductive status. These genes might provide insights into brain changes necessary to initiate and maintain oriented flight activity.

We tried to ensure that the animals used for the microarray analyses were handled in a way to minimize the influence of non-migratory factors on gene expression and to mimic the conditions used for our behavioral experiments (Table 1). First, all animals were placed in glassine envelopes to minimize the influence of activity on the array analysis (number of days each group was in the envelopes is depicted in Table 1). Second, all the animals were housed in controlled environmental conditions simulating those in the outside environment at the time of year of collection, as outlined in Table 1; all butterflies were collected in the wild and the locales and times of year of collection were recorded (Table 1). Third, the environmental conditions within the compartments in which the butterflies were housed (lighting cycle, temperature and humidity) were similar to those used to generate the behavioral data for the different groups shown in Figures 1 to 5. Fourth, to minimize time-of-day effects on gene expression, all animals were killed within a 2-hr period encompassing the mid-light time of the light-dark cycle in which the various butterfly groups were housed.

**Table 1:** Animal Characteristics

			Reproductive Status			
					
Category	ID*	Sex	Male (glands weight, g)	Female (# of mature oocytes)	Source/Date Collected	Housing Conditions
	**S**-M03		0.0217			
	**S**-M04		0.0223			
	**S**-M07	Male	0.0218		Near	2 days LD cycle
	**S**-M10		0.0313		Greenfield,	Lights on: 4:38 AM
Summer (S)	**S**-M11		0.0259		Massachusetts	Lights off: 7:09 PM
					(Lat 42°59'N)	Temperature: 20°C
	**S**-F19			70	(Long 72°60'W)	Humidity: 70%
	**S**-F20			88	7/30/07	
	**S**-F21	Female		> 100		
	**S**-F22			> 100		
	**S**-F23			> 100		

	**F**-M07		0.0084		Near	2 days LD cycle
	**F**-M10		0.0056		Port Lavaca,	Lights on: 6:09 AM
	**F**-M23	Male	0.0087		Texas	Lights off: 4:54 PM
	**F**-M29		0.0070		(Lat 28°36'N)	Temperature: 20°C
Fall (F)	**F**-M32		0.0065		(Long 96°37'W)	Humidity: 70%
					10/30/07	
	**F**-F03			0		
	**F**-F13			0		
	**F**-F14	Female		0		
	**F**-F18			0		
	**F**-F21			0		
		
	**M**-M03		0.0357			14 days LD cycle
	**M**-M04		0.0557			Lights on: 6:00 AM
	**M**-M08	Male	0.0341			Lights off: 5:00 PM
Fall methoprene (M)	**M**-M13		0.0559			Temperature (light):
	**M**-M15		0.0578			23°C
						Temperature (dark):
	**M**-F20			> 100		12°C
	**M**-F27			> 100		Humidity: 70%
	**M**-F30	Female		> 100		
	**M**-F23			> 100		
	**M**-F24			> 100		
		
	**V**-M10		0.0179			
	**V**-M13		0.0198			
	**V**-M14	Male	0.0117			
Fall vehicle (V)	**V**-M18		0.0121			
	**V**-M19		0.0106			
			
	**V**-F04			0		
	**V**-F22			0		
	**V**-F24	Female		0		
	**V**-F27			0		
	**V**-F29			0		

*Animal ID code: **Group(S, F, M or V)**-sex (M or F) animal number

We collected total brain RNA from 10 summer butterflies, 10 fall migrants, 10 migrants following methoprene treatment, and 10 migrants following vehicle (acetone) treatment. We checked the reproductive status of all animals to ensure they had the expected reproductive state (Table 1), which was similar to that found in our flight studies (Figure 4A); summer monarchs and methoprene-treated migrants had activated reproductive systems, while untreated and vehicle-treated migrants did not (Table 1). The brain RNAs were amplified and then used to probe a custom Nimblegen array that was designed to analyze the 9417 unique cDNA sequences established in our published brain EST library [11].

To discover genes that might be involved in oriented flight, but not reproduction, we compared the summer group with each of the three fall groups (untreated, methoprene-treated, and vehicle-treated) for males and for females, and looked for gene regulation patterns common among the three comparisons for each sex (Table 2A; see Additional file 1 for specific ESTs and annotation). The rationale for this approach was that methoprene would only regulate JH-dependent genes and should not affect genes that regulate directional flight and orientation, as our flight experiments showed that oriented flight was not altered by increasing JH activity (Figures 2 and 4).

**Table 2:** Statistical Comparisons

A. Orientation Genes				
Sex	Comparison Test*	# of significant	# shared in each sex+	# shared by both sexes

	Summer vs. Fall	1009		
Male	Summer vs. Fall methoprene	985	410	
	Summer vs. Fall vehicle	3832		40
	
	Summer vs. Fall	995		
Female	Summer vs. Fall methoprene	960	212	
	Summer vs. Fall vehicle	1465		

B. JH-response Genes				

Sex	Comparison Test*	# of significant	# shared in each sex†	# shared by both sexes

Male	Summer vs. Fall	1009	270	
	Fall methoprene vs. Fall vehicle	3122		23
	
Female	Summer vs. Fall	995	115	
	Fall methoprene vs. Fall vehicle	1195		

*All statistical tests were done using student's *t *test with the numbers of significant genes (*p *< 0.05) shown.

+ See Additional file 1 for specific ESTs and annotation.

† See Additional file 2 for specific ESTs and annotation.

**Figure 6:**
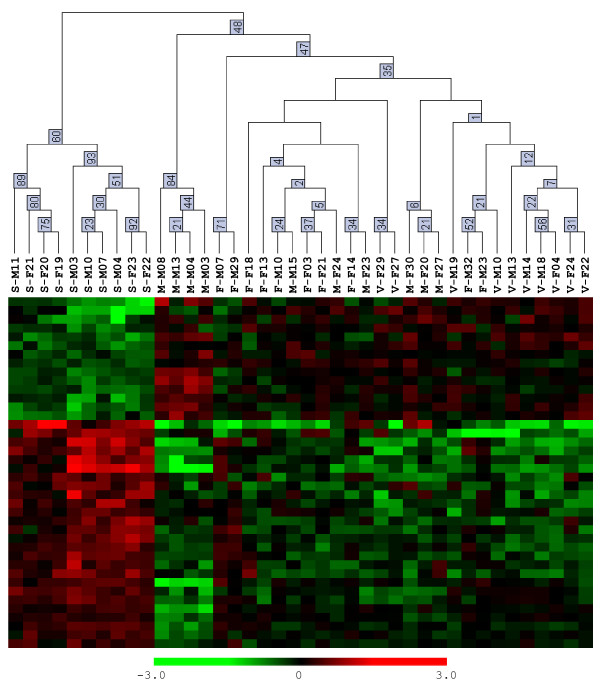
**Clustering of orientation genes**. Hierarchical clustering of individual butterflies (top) based on expression profiles of the 40 regulated genes expressed as heat maps (below). Animal ID code for clustering is as depicted in Table 1. Bootstrap analysis provides confidence values at the nodes. The heat maps show estimated gene expression levels (red upregulated; green downregulated). The genes have been ordered according to *k*-means clustering.

As the comparisons were done separately for males and females, and our behavioral data did not show significant sex differences in flight directionality and orientation (Figure 3), we focused on the common differentially regulated genes that were shared between males and females (Table 2A, right column). Accordingly, we identified 40 cDNAs that were differentially regulated between summer butterflies and fall migrants, irrespective of sex. Furthermore, hierarchical clustering analysis using the individual animal expression data for only these 40 cDNAs showed that all 10 summer animals were clustered together correctly, whereas the three fall groups formed their own separate cluster; bootstrap values supported this trend (Figure 6). The expression of 14 genes was increased in migrants, while the expression of 26 genes was increased in summer butterflies (Figure 6, listed from top to bottom of heat map, respectively). The magnitude of the differences was uniformly modest, with a mean expression difference among the 40 genes of 1.70-fold (range = 1.28-fold to 3.21-fold). Nonetheless, the expression profile of these 40 genes appears to predict a critical behavioral characteristic of fall migrants in individual butterflies – oriented flight behavior, independent of reproductive state. It is important to note, however, that some of the differences in gene expression could reflect non-migratory aspects of monarch biology that were hard to control in our studies, including the age of the butterflies and the mixed genetic background inherent in collecting wild animals.

**Figure 7:**
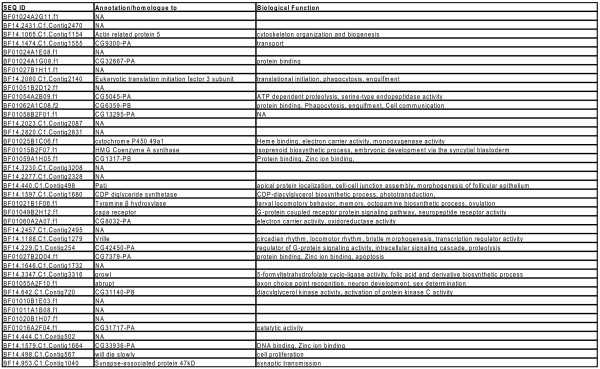
**Annotation of orientation genes**. The sequence ID, annotation, and Biological Function based on gene ontology (GO) of the orientation genes are shown. They are listed in the same order they are represented on the vertical axis of Figure 6. NA means the gene is either not annotated or annotated but without GO function assigned.

Of the 40 cDNAs, only 25 had matches with other databases, with 24 being annotated with function (Figure 7). The annotated brain cDNAs that were upregulated in migrants included those involved in cytoskeletal organization (*Actin related protein 5*), ATP dependent proteolysis (*CG5045*), immune responses (*Eukaryotic translation initiation factor 3 subunit *and *CG6359*) and the initiation of translation (*Eukaryotic translation initiation factor 3 subunit*). The annotated cDNAs that were upregulated in summer butterflies included those that may be involved in neural and behavioral plasticity, including those involved in neuronal development (*abrupt*), synaptic transmission (*Synapse-associated protein 47 kD*), and cell proliferation (*will die slowly*).

The two differentially regulated cDNAs that appeared to be most directly related to time-compensated orientation were *tyramine beta hydroxylase*, whose protein regulates octopamine biosynthesis, which is involved in motor behavior, and *vrille*, which encodes a circadian clock component (based on studies in *Drosophila*); both cDNAs were upregulated in summer butterflies. The protein VRILLE is an important negative regulator of *Clock *transcription, and CLOCK is a critical transcriptional regulator of the circadian clock mechanism of insects and mammals [13].

There were also several differentially regulated cDNAs, upregulated in summer butterflies, that were involved in more general cellular processes, which included steroid/cholesterol metabolism (*Cytochrome P450 *and *HMG Coenzyme A synthase*), lipid metabolism (*CDP-diglyceride synthetase *and *CG31140-PB*), electron transport (*Cytochrome P450 *and *CG8032-PA*), and intracellular signaling pathways (*growl*, *capa receptor*, and *CG42450-PA*).

The one annotated cDNA without assigned function can now be classified as being involved functionally in oriented flight behavior, along with the other 39 cDNAs. Interestingly, 15 cDNAs had no annotation with other databases. Their lack of identity based on available genomic and EST resources could mean that the non-annotated cDNAs contain incomplete sequence information for orthologous matches with other databases. A more exciting possibility is that the non-annotated cDNAs represent unknown genes whose functions are unique to the migratory state in monarch butterflies.

### JH-regulated gene expression patterns in brain correlate with reproductive state

In addition to 'orientation' genes, we were also interested in evaluating the JH-response genes. These genes are likely involved in reproductive status and longevity. Since these genes are expected to be regulated by JH, the methoprene-treated fall butterflies should have expression profiles similar to those in the summer animals. Again, we performed sex-specific statistical analyses, and compared the summer and the fall groups, and the methoprene-treated and vehicle-treated migrants (Table 2B). We then screened for shared genes between the two groups for each sex.

**Figure 8:**
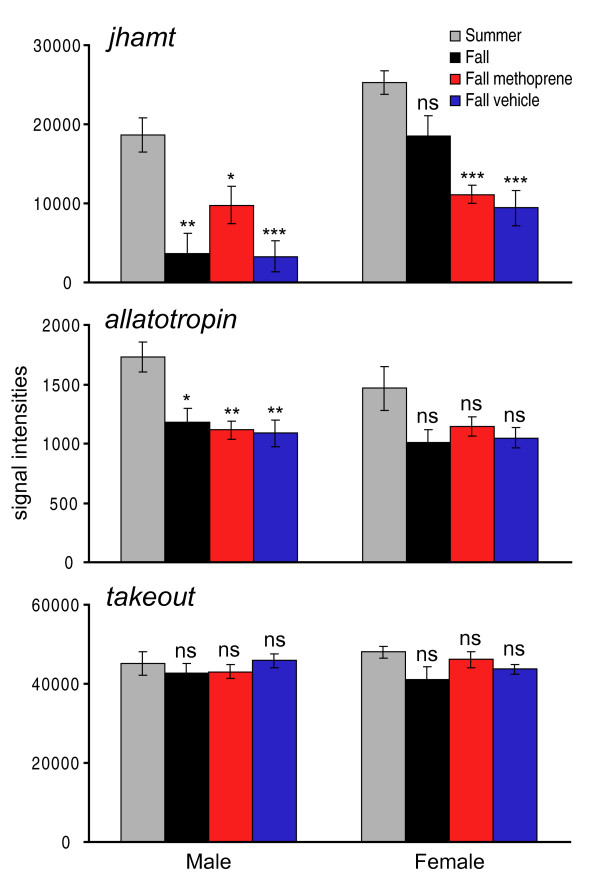
**Comparison of sex-specific microarray expression profiles of *juvenile hormone acid methyltransferase *(*jhamt*), *allatotropin*, and *takeout***. The profiles were compared across the four experimental groups. For each sex, each of the three fall groups was compared with summer, using unpaired 2-tail *t *test (with unequal variance). ns, not significant, *p *> 0.05; * *p *< 0.05; ** *p *< 0.01; *** *p *< 0.001.

Of the sex-specific groups of differentially regulated genes (Table 2B, # shared in each sex; for complete list of JH-regulated, sex-specific ESTs and annotation see Additional File 2), we focused on three genes involved in increased JH activity for which we previously showed significant increased expression in summer butterflies (compared with fall migrants) by real-time polymerase chain reaction (qPCR): *juvenile hormone acid methyltransferase (jhamt)*, which encodes the enzyme that mediates the last step in JH biosynthesis [14];*allatotropin*, which encodes a neuropeptide that can increase JH synthesis [15]; and *takeout*, which encodes a potential JH-binding protein [16]. The mRNA levels for both *jhamt *and *allatotropin *were upregulated significantly in summer males compared with untreated male migrants by microarray; mRNA levels were also higher in female summer butterflies but the levels did not reach significance (Figure 8). Combining the sexes and reanalyzing the microarray differences between summer butterflies and untreated migrants showed significant up-regulation of both *jhamt *and *allatotropin *expression in summer butterflies (*p *< 0.01), which was consistent with our previous qPCR results, as animals of mixed sex were used in that analysis [11]; as both *jhamt *and *allatotropin *are associated with the corpora allata, it is likely that the expression profiles of each represent expression mainly in corporal allata tissue, which was likely included in our brain dissections. The mRNA levels of *takeout *were marginally upregulated in summer butterflies by microarray analysis (Figure 8), but were not upregulated significantly, as those reported by qPCR in our previous work [11]. It is noteworthy that the qPCR results were performed on mRNA from whole heads, while the microarray analysis was performed using mRNA from dissected brains; this disparity in tissue composition likely contributed to any expression discrepancies between the previous qPCR study and our current microarray results.

**Figure 9:**
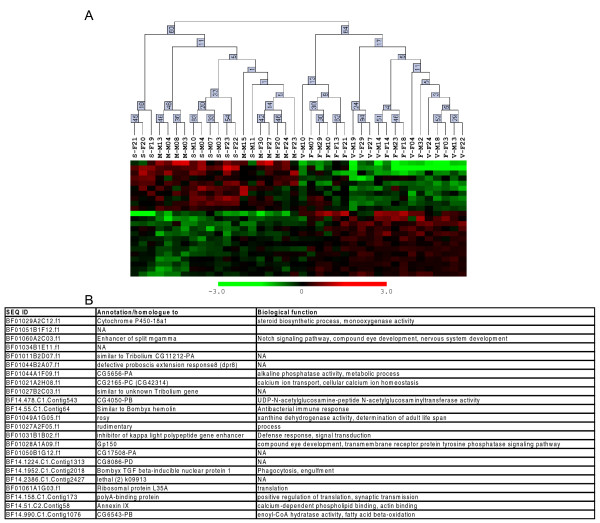
**Cluster and annotation of JH-response genes**. (A) Hierarchical clustering of individual butterflies (top) based on expression profiles of the 23 regulated genes expressed as heat maps (below). Animal ID code for clustering is as depicted in Table 1. Bootstrap analysis provides confidence values at the nodes. The heat maps show estimated gene expression levels (red upregulated; green downregulated). The genes have been ordered according to *k*-means clustering. (B) The sequence ID, annotation, and Biological Function based on gene ontology (GO) are shown below. They are listed in the same order they are represented on the vertical axis of panel A. NA means the gene is either not annotated or annotated but without GO function assigned.

We next examined cDNAs that were differently regulated in both males and females (Table 2B, # shared by both sexes), to determine whether we could identify JH-regulated genes involved in more global processes that would not be expected to be sex-specific, such as longevity and fatty acid metabolism. We identified 23 putative JH-response genes that were common between the males and females. Hierarchical clustering using the 23 JH-regulated genes showed that summer and methoprene-treated migrants clustered together, as expected (Figure 9A). The other two groups, the untreated and vehicle-treated migrants, formed their own cluster (Figure 9A). The expression of 11 genes was increased in the summer and methoprene-treated migrants, while the expression of 13 genes was increased in the untreated and vehicle-treated migrants (Figure 9A, listed from top to bottom of heat map, respectively). Similar to the orientation genes, the magnitude of the differences was uniformly modest for the JH-regulated genes, with a mean expression difference among the 23 genes of 1.67-fold (range = 1.24-fold to 3.28-fold). Thus, the hierarchical clustering predicted correctly the reproductive status of all individual animals tested, using the brain expression pattern of these 23 genes that are common between the sexes.

Of the 23 JH-related cDNAs, 21 had matches with other databases, with 15 annotated with biological function (Figure 9B). Predictably, they included genes involved in longevity (*rosy*), fatty acid metabolism (*CG6543-PB*), and, interestingly, immune responses (*hemolin*, *TGF beta-inducible nuclear protein 1*, *Gp150*, and *inhibitor of kappa light polypeptide gene enhancer*), which were all upregulated in untreated and vehicle-treated migrants. There were also two genes involved in translation (*ribosomal protein L35A *and *polyA-binding proteins*) and one involved in calcium-dependent phospholipid binding (*Annexin IX*) that were upregulated in JH-deficient migrants. Genes that were upregulated in reproductive butterflies included those involved in steroid biosynthesis (*Cytochrome P450-18a1*), notch signaling (*Enhancer of spilt mgamma*), and calcium homeostasis (*CG2165-PC*).

The six cDNAs that were annotated but without assigned function can now be classified functionally as being involved in JH-related activities, along with the other 17 cDNAs. Two differentially regulated cDNAs lacked annotation based on available genomic and EST databases, which could mean that they contain incomplete sequence information for orthologous matches with other databases or that the non-annotated cDNAs represent unknown genes whose functions are unique to the migratory state in monarch butterflies.

### Defining the migratory state

The thrust of this work was to more precisely define the behavioral and molecular differences between summer butterflies and fall migrants. As there are several generations of reproductively active spring and summer butterflies, we chose to focus on the mid- to late-summer butterflies whose offspring likely give rise to fall migrants. We contrast this generation with those in the spring and early summer, which are moving north/northwesterly to repopulate the upper ranges of their habitat in Eastern North America. Observations of those monarchs suggest that they may have oriented flight behavior [7], and these earlier generations need to be more rigorously evaluated in a flight simulator, as we have done in our studies with mid- to late-summer butterflies.

A significant aspect of our behavioral work with summer butterflies shows that their individual flight patterns are uniformly non-directional. A prior study using the disappearance bearing of released monarchs showed that summer butterflies collected at a similar time of year as ours (early August at latitude 38'9°N) were not significantly oriented [10]. Only short flight paths can be assessed in the disappearance bearing studies, but the results are consistent with our longer flight recordings in a flight simulator. This non-directionality is an important behavioral trait that characterizes mid- to late-summer butterflies from the other generations that occur over the course of the year. These animals also provide the clearest behavioral difference with fall migrants, as fall migrants consistently exhibit directional flight, which is why we used them in our gene expression studies.

Another distinguishing feature between summer butterflies and fall migrants is reproductive state. Summer butterflies are reproductively competent, while fall migrants are JH deficient, which leads to reproductive diapause, with decreased weight of reproductive organs and quiescent reproductive activity [2,3]. Reproductive diapause usually persists over the course of the migratory journey and for months at the overwintering sites [8]. However, reproductive diapause can be readily manipulated in fall migrants; diapause can be broken by exposing migrants to elevated temperatures and increasing day length [10,17]. It is unclear whether the entire repertoire of migratory behaviors (including reproductive diapause and directional flight behavior) are initiated by the same environmental cues, which may include decreasing day length, sun angle and temperature, and the age of the larval food source [18].

Our studies show clearly that directional flight activity and time-compensated sun compass orientation persist independent of reproductive state; oriented flight activity has also been shown in disappearance bearing studies of reproductively active migrants [10], but persistent time-compensation had not been shown previously. It is still possible that JH deficiency is involved in the induction of directed flight for sun compass orientation, but it is clear from our results that persistent JH deficiency is not required for maintenance. Moreover, increasing JH activity in migrants is unlikely to explain the reversed flight direction of migrants (in the northerly direction) as they leave the overwintering grounds in the spring, because the methoprene-induced increase in reproductive activity in fall migrants did not alter the normal south/southwesterly flight direction manifested when studied in LD (Figure 2A). It will be interesting to determine in future experiments whether JH antagonist treatment can convert summer butterflies to migrants.

Consistent with our findings of persistent migratory flight in reproductive migrants, ecological observations have suggested that a small number of migrating monarchs, who have broken reproductive diapause because of prolonged exposure to high environmental temperatures during their migration south, may give rise to a subsequent 'backup' generation of migrants, originating from the southern range late in the fall (See [19]). Indeed, a peak in monarch egg and larva abundance in Texas during September and early October supports this idea, because adult monarchs are not seen in the southern United States throughout most of the summer [20].

An exciting aspect of our work was the discovery of a suite of 40 genes whose differential expression in brain distinguishes individual summer butterflies from fall migrants, independent of reproductive status. Any single gene or combination of genes within the 40 could be essential for the initiation and/or maintenance of directional flight in migrants. The genes that were not annotated may be especially interesting targets for further studies, as we have shown their importance in predicting oriented flight. Although the fold changes in gene expression between non-oriented and oriented butterflies were small, they do not rule out larger differences in expression of individual genes in specific neural subpopulations that have been diluted by whole brain analysis [21] – a possibility that needs to be evaluated for each gene. We expect that with further study the number of differentially regulated genes will grow, as the sequencing and annotation of the entire monarch genome progresses.

## Conclusion

Our data are the first to provide a link between alterations in gene expression profiles in brain and migratory state in any animal which undergoes long-distance migration. Moreover, our results also provide the first insights into gene expression patterns in brain that may underlie time-compensated sun compass orientation, a complex process involving brain integration of information about time and space.

Our gene expression profiles resemble those reported to be involved in behavioral plasticity in honey bees in which a small collection of genes, most of which did not show a greater than 2-fold change by microarray analysis, reliably predict behavioral state (nurses from foragers) in individual bees [21]. Further evaluation of the 40 genes we have identified in monarchs will likely provide novel insights into their individual and/or collective importance for migration and the brain changes necessary to initiate and maintain oriented flight behavior.

## Methods

### Animal housing

Monarch butterflies were housed in the laboratory in glassine envelopes in Percival incubators with controlled temperature, humidity (70%), and lighting. The butterflies were fed 25% honey every other or every third day.

### Methoprene/vehicle treatments

Animals were treated topically on their abdomens with either 200 μg of methoprene in 5 μl acetone or acetone alone on day 1 and day 3.

### Evaluation of reproductive status

For males, the ejaculatory duct and tubular gland were weighed. For females, the presence of mature oocytes was recorded.

### Flight analysis

Butterflies were tethered as previously described [5], and flight behavior was monitored using a modified Mouritsen and Frost flight simulator as described [22]. Butterflies were flown outdoors under sunny skies when the sun could be seen from their position in the flight barrel. Data were analyzed to determine the significance of orientation and the mean direction using circular statistics [23].

### Microarray sample preparation

Over a 2-hour period bracketing (± 1 hr) the middle of the light period, individual monarchs were taken from their envelopes, and the heads were removed with scissors. The severed heads were immediately placed in 0.5× RNAlater (Ambion), and each brain was dissected, with the photoreceptor layer removed, and placed on dry ice; brains were stored at -80°C. RNA from individual brains was isolated using the Qiagen RNeasy Mini Kit with the optional on-column DNase treatment according to the manufacturer's instruction. Approximately 100 ng RNA from each brain sample was used to synthesize amplified cDNA using the Ovation Amplification System V2 (Nugen). Amplified cDNAs were purified using Qiagen PCR purification kit. All 40 samples were successfully amplified with yields between 10 and 15 μg of cDNA. As required by Nimblegen microarray service, 6 μg of amplified cDNA from each sample were converted into doublestrand cDNA using Klenow (NEB) reaction with random hexamer priming (Promega). Doublestrand cDNAs were purified again using Qiagen PCR purification kit before submitting to Nimblegen for labeling and hybridization.

### Microarray design and analysis

A 4-plex Nimblegen custom monarch expression array was designed based on our previously published EST data to include all unique ESTs and contigs. In addition, 18 previously cloned monarch genes were included on the array. A total of 9417 unique sequences were incorporated into the array design. Each sequence was represented by seven non-overlapping oligos on the array. Nimblegen's service department carried out array design, synthesis, probe labeling and hybridization. They also performed data pre-processing including array scan, data extraction and normalization [24]. Normalized values for each gene, which provide a measure of expression levels, were used in all data analyses. All normalized values and processed data are available at GEOdatabase .

### Data analysis

To determine if gene expression levels differed between butterflies in different behavioral states, we used a Student's *t*-test to compare normalized values for each gene between treatment groups (Table 2). We compared differences in gene expression levels at the conventional value of *p *< 0.05. Although the use of an unadjusted significance level may increase false positives due to multiple testing, the number should be minimal in our case, because results were drawn from comparisons of shared gene across multiple groups and not a single comparison (see Table 2). For those genes that showed a significant change in expression level, we labeled them as either 'upregulated' or 'downregulated' depending on the means for each group. For example, if the mean normalized value was higher in the summer group than the migrant group, then the gene was considered upregulated in summer butterflies. Statistical comparisons and gene cluster analyses were done using ArrayStar software. Animal cluster analyses were done using MultiExperiment Viewer . Gene annotation was done using the previously published ESTIMA monarch EST database .

## Competing interests

The authors declare that they have no competing interests.

## Authors' contributions

All authors contributed to experimental design, execution, data analysis and writing the paper. All authors have read and approved the final manuscript.

## Supplementary Material

Additional file 1 **Sex-specific orientation genes.** Click here for file

Additional file 2 **Sex-specific juvenile hormone-regulated genes.** Click here for file
